# Magnetic Photocatalyst Nanocomposite Based on MnFe_2_O_4_@ZnO for AZO Dye Degradation

**DOI:** 10.1021/acsomega.4c11468

**Published:** 2025-04-26

**Authors:** Javier Alonso Lopez Medina, David Domínguez, Pedro Pizá, Guoduan Liu, Camilo Velez, Faustino Reyes Gómez, Mario Humberto Farías, Uriel Caudillo-Flores, Gerardo Soto Herrera, Hugo Tiznado, Jorge Ricardo Mejía-Salazar

**Affiliations:** † SECIHTI - IxM - Centro de Nanociencias y Nanotecnología, Universidad Nacional Autónoma de México, Ensenada, B.C. C.P. 22800, México; ‡ Centro de Nanociencias y Nanotecnología, Universidad Nacional Autónoma de México, Ensenada, B.C. C.P. 22800, México; § Centro de Investigación en Materiales Avanzados−CIMAV, Chihuahua, CH C.P. 31136, México; ∥ Materials and Manufacturing Technology- University of California, Irvine, California C.P. 92697-3975, United States; ⊥ Department of Mechanical and Aerospace Engineering, University of California, Irvine, California C.P. 92697-3975, United States; # National Institute of Telecommunications (Inatel), Santa Rita do Sapucaí C.P. 37536-001, Brazil

## Abstract

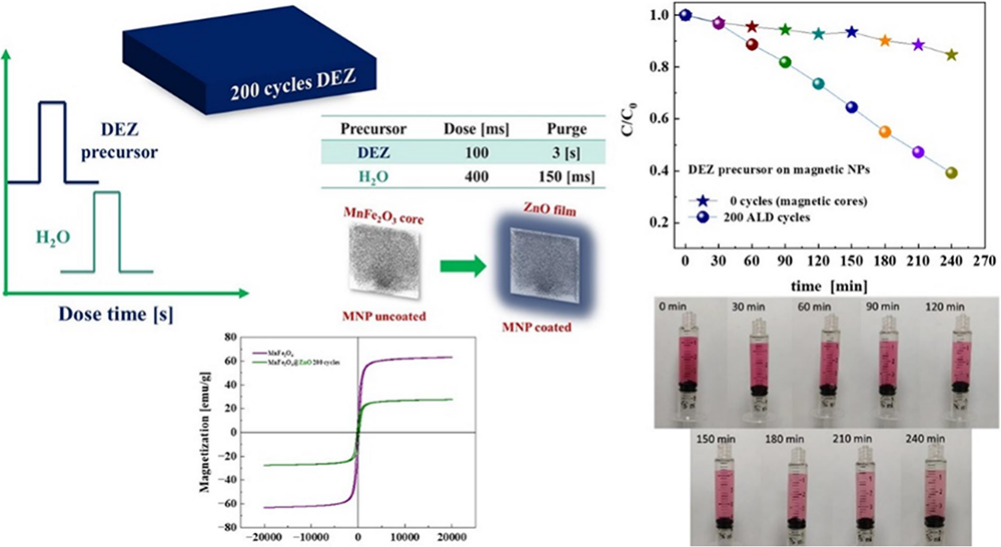

This work focuses on fabricating a photocatalyst nanocomposite based on MnFe_2_O_4_@ZnO for degrading Red Amaranth azo dye. Manganese ferrite (MnFe_2_O_4_) magnetic nanoparticles were synthesized via a hydrothermal method, while a ZnO thin film, acting as the photoactive layer, was deposited on the magnetic cores using the atomic layer deposition (ALD) technique. X-ray diffraction (XRD) confirmed the spinel ferrite structure of MnFe_2_O_4_ and the hexagonal wurtzite phase of ZnO. The crystallite size, determined from the (311) peak, was 36.5 nm; this value was consistent with the average size of 33.2 nm measured by transmission electron microscopy (TEM). Magnetic characterization via vibrating sample magnetometry (VSM) at room temperature revealed a superparamagnetic behavior, determined by a very small hysteresis loop. The ZnO coating, achieved with 200 ALD cycles, resulted in a degradation efficiency η_eff_ of approximately 60% for the Red Amaranth dye. Finite-difference time-domain (FDTD) simulations provided theoretical insights into the electromagnetic interactions driving the photodegradation process, supporting the UV–vis absorbance data of the AZO dye. This nanocomposite can be considered as a soft magnetic material that offers promising applications in nanotechnology for environmentally friendly wastewater treatment and remediation.

## 1 Introduction

Currently, rapid population growth and industrialization have caused serious impacts on the environment and human health in countries where environmental regulation policies are scarce or are not applied according to established laws. For instance, wastewater generated by dye, food, cosmetic, textile, and pharmaceutical industries discharge harmful effluents directly into the ecosystem without control.^1,2^ These effluents carry numerous toxic organic substances, causing various detrimental effects on the environment, flora, and aquatic fauna.^3^ These effects can have significant consequences, such as reduction in dissolved oxygen levels in water, formation of persistent and toxic compounds harmful to aquatic organisms living in freshwater sources used for human consumption, obstruction of light penetration into the water bodies, among others.^4,5^ Furthermore, organic pollutants can also harm human health by being carcinogenic, teratogenic, mutagenic, and interfering with the endocrine system.^6−13^ Nowadays, there are multiple techniques applied in the decontamination of wastewater, including electrodialysis, membrane filtration, precipitation, adsorption, electrochemical reduction, and electro-deionization.^14−20^ However, these processes often consume large amounts of energy and present complications in transferring contaminants between different fluids and generating byproducts during treatment.^21,22^ Therefore, it is of great importance to develop environmentally friendly, cost-effective, and highly efficient methods for wastewater treatment. For instance, heterogeneous photocatalysis has been widely studied and applied in various areas such as water purification, CO_2_ reduction, and N_2_ fixation. This method can degrade organic contaminants in wastewater and transform them into carbon dioxide, water, or other small molecules.^22−28^

It is known that nanotechnology plays an important role in generating knowledge at the applied level to resolve real problems that affect society, especially in vulnerable populations exposed to contaminated water consumption.^29,30^ Thus, the new knowledge involves understanding different topics related to synthesis processes in new materials, modifying and controlling their physical and chemical properties, and their potential applications according to the needs of the industry and society. With the knowledge gained in fabrication methods, nanostructures have been prepared for specific desired applications by controlling their magnetic, electrical, optical, thermal, electronic, or mechanical properties.^31−33^ These types of nanostructures can be referred to as “functional nanostructures”, and the stimulus-response relationship is key in determining the field of knowledge in which they can be employed. In this context, there is concern about modifying the physical and chemical properties of nanostructures when combining materials of different nature or behavior, generating more than one physical response in the material.^34−37^ The combination of magnetic properties of magnetic cores with the optical response associated with a semiconductor material allows the development of so-called magnetically controlled multifunctional materials that can be applied in fields such as photocatalysis for water treatment.^38−41^ The magnetic properties of different materials can be of particular interest due to the possibilities of external manipulation by the application of external magnetic fields. This is the case with spinel-type ferrites MFe_2_O_4_, where M can be a transition element (a cation of Co, Ni, Fe, Mn, or Zn with different stoichiometry). These ferrites are characterized by high chemical stability, magnetic behavior intimately linked to their dimensionality, biocompatibility, and their ability to absorb or emit radiation over a wide range of the electromagnetic spectrum (UV–vis-NIR).^42,43^ Such ferrites have exhibited interesting properties as multifunctional materials when these are combined with a photoactive material that allows them to be used in photocatalytic tests to degrade contaminants and to be able to be separated magnetically with an external field using a conventional magnet.^38,44−47^ In addition, multiple types of catalysts can be employed in photocatalytic processes based on ferrites with different crystal structures, such as spinel, garnet, magnetoplumbite, or orthoferrite. These kinds of materials are characterized by being oxides containing Fe^3+^ and at least another metal cation (transition metal), which have been applied as powders, films, or ceramics. The photocatalyst based on magnetic ferrites has attracted great interest and has quickly become a trending topic among scientists, as reflected in the increasing number of publications per year in the last 8 years, from less than 10 to more than 40.^48−50^

Many researchers have utilized ferrite photocatalysts for degrading organic pollutants. Ramadoss et al., in 2020,^51^ synthesized magnetic nanoparticles of MnFe_2_O_4_ for the photocatalytic degradation of Congo Red dye under visible light. In degradation studies conducted using a photoreactor with irradiation in the range of 565 nm, approximately 98.3% of the dye was successfully mineralized and transformed into nontoxic alcohols and acids.^52^ Furthermore, due to the magnetic nature of the nanoparticles, they could be easily recovered and utilized in multiple cycles. Taghavi Fardood et al., in 2019,^53^ tried the malachite green dye photodegradation from the synthesis of magnetic nanoparticles based on MgFe_2_O_4_. They found that the obtained nanoparticles exhibited superparamagnetic behavior as well as high photocatalytic activity under visible light irradiation. They were able to degrade the malachite green dye up to 98% and took advantage of the magnetic response of the ferrite to carry out different repetition cycles, recycling the nanoparticles through magnetic separation processes. Rodiah and Ditiyaningrum, in 2022,^54^ performed the green synthesis of a photocatalyst based on NiCoFe_2_O_4_ nanoparticles. They used these nanoparticles to perform photodegradation for 300 min under UV light of diazinon, an organophosphate insecticide used to control insects in the soil, in ornamental plants, and fruit and vegetable crops, reaching a percentage degradation of 72.4% using 12 mg of catalyst.

Wang et al., in 2016,^55^ studied the photodegradation of Rhodamine B dye using magnetic nanoparticles of Fe_3_O_4_ coated with ZnO as a core–shell structure catalyst. In this case, after Fe_3_O_4_ nanoparticles were synthesized via the chemical coprecipitation method, a ZnO coating was added through the hydrothermal method. They found that this core–shell structure in aqueous solution under ultraviolet light achieved a 99.3% degradation of Rhodamine B, taking only 60 min. Also, they found that larger-sized nanoparticles presented better catalytic activity. This was attributed to the fact that the concentration of surface oxygen vacancies is a vital factor for photocatalytic performance. The advantage of the core–shell structure was highlighted, as it exhibited a higher percentage of efficiency in dye degradation compared to using only photocatalytic material, in addition to facilitating its recovery and subsequent reuse.

**Figure 1:**
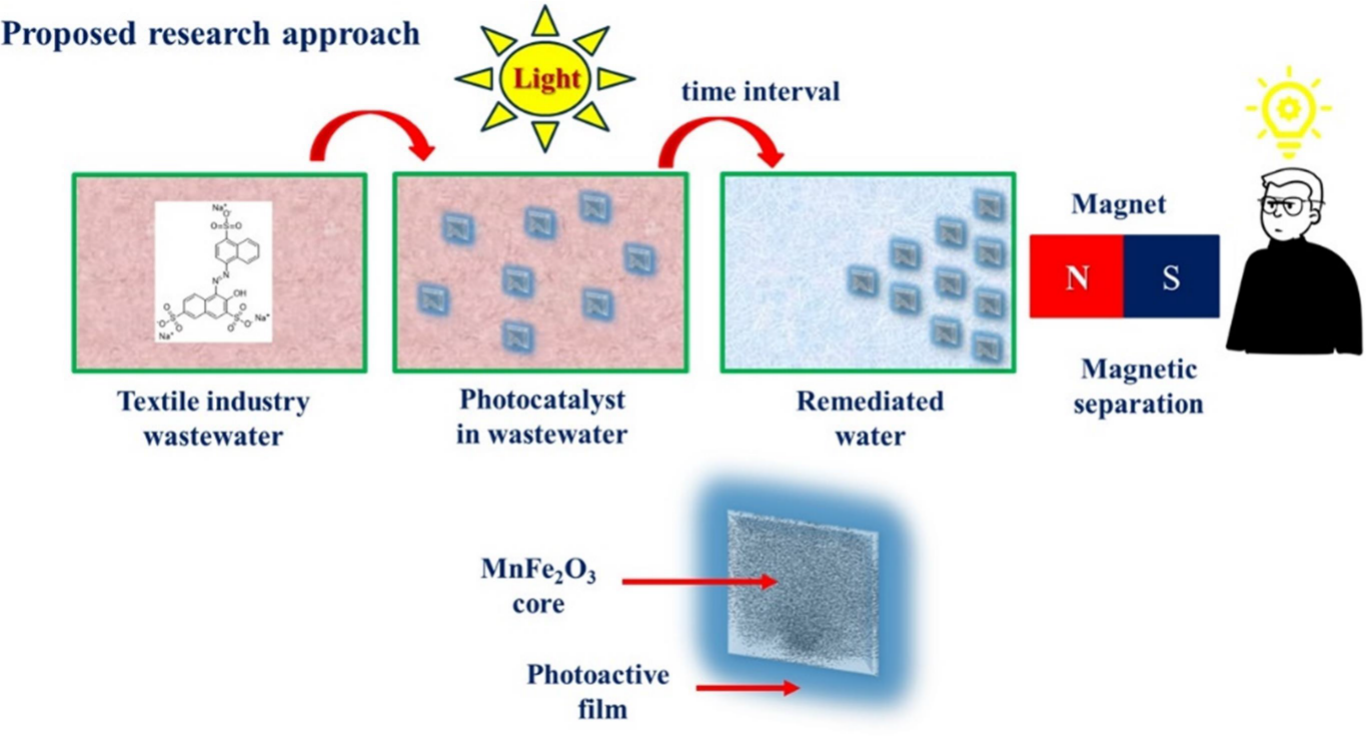
Schematic overview of the proposed research approach.

In this work, we consider the problems of the presence of azoic dye in wastewater that is discharged by the textile, food, dye, and pharmaceutical industries. Photoactive ZnO thin films with different thickness values were grown over magnetic nanoparticles via atomic layer deposition (ALD). Since one of the objectives was to prepare a nanocomposite material that could be easily recovered from aqueous solutions using a conventional magnet, as shown in Figure 1, the magnetic core photocatalyst MnFe_2_O_4_@ZnO was prepared via a hydrothermal method for AZO dye degradation, combining two synthesis techniques in synergy to create a multifunctional material. This material integrates the magnetic properties of ferrites with the photoactive characteristics provided by a ZnO thin film coating.^38^ Studied samples were widely characterized and applied in the photocatalytic degradation of toxic Red Amaranth azo dye.

## 2 Experimental Section

### 2.1 Magnetic Core Photocatalyst Fabrication

**Figure 2:**
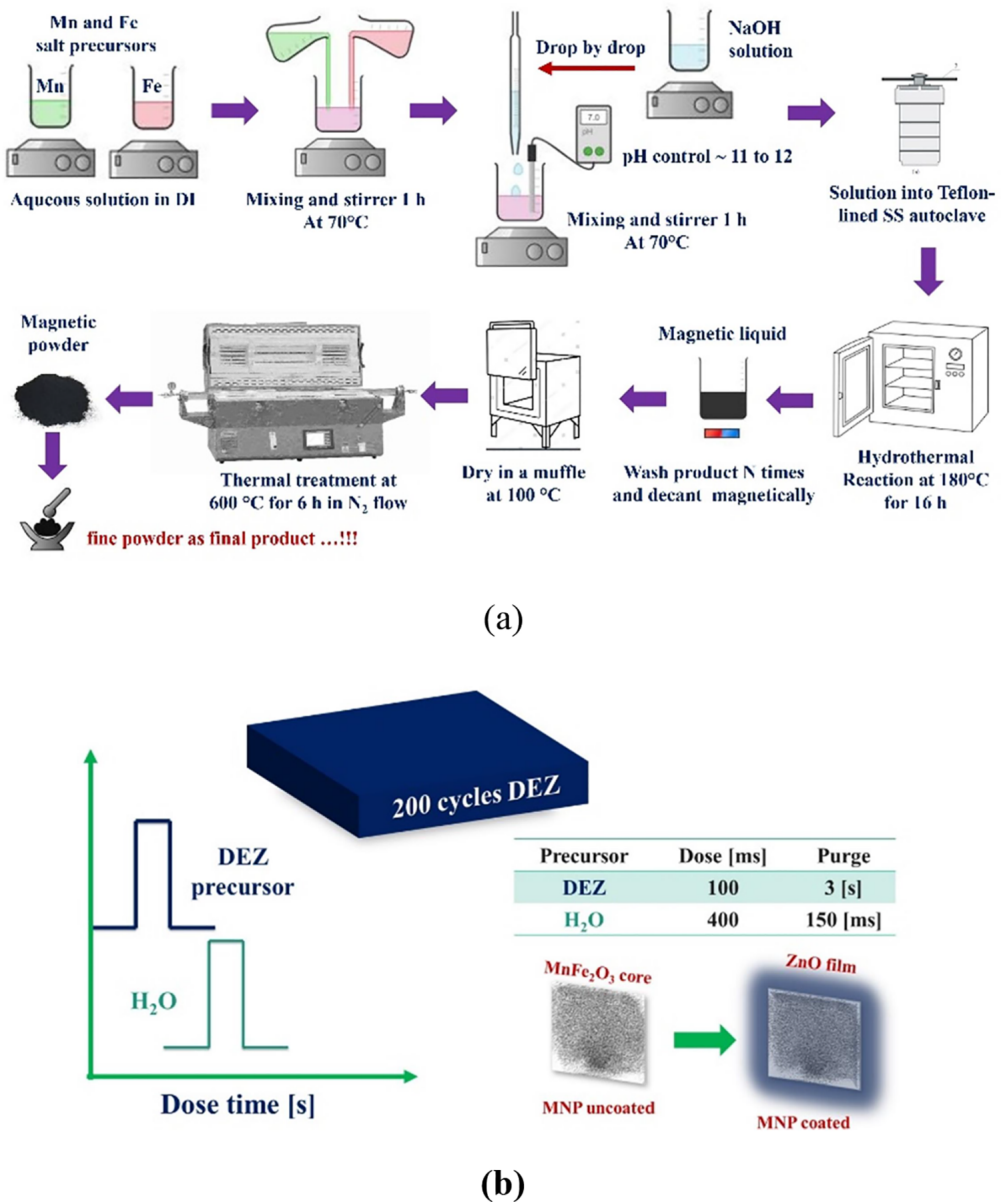
Nanoomposite material scheme fabrication (a) MnFe_2_O_4_ magnetic cores synthesis by hydrothermal method and (b) MnFe_2_O_4_@ZnO nanocomposite by the ALD process to be coated with ZnO as the photoactive material on the magnetic cores surface.

The preparation of the nanocomposite material involves two main stages, each meticulously optimized to achieve the desired properties and performance in the final product. First, MnFe_2_O_4_ magnetic powders are synthesized via the hydrothermal method, where precursor materials are exposed to controlled temperature and pressure within a hydrothermal reactor,^56,57^ forming the desired magnetic powder.^58^ Next, a ZnO layer is applied to the magnetic nanoparticles using ALD.^59−61^ This technique allows for highly precise, atomic-level conformal coatings, resulting in a uniform ZnO layer on the MnFe_2_O_4_ nanoparticles. As ZnO is a well-known semiconductor with photocatalytic properties,^62^ this layer enhances the nanocomposite’s functionality. Figure 2a,b provides a schematic overview of the fabrication process for MnFe_2_O_4_ magnetic nanoparticles and MnFe_2_O_4_@ZnO nanocomposites.

#### 2.1.1 Magnetic Nanoparticle Synthesis

The magnetic cores were fabricated using the hydrothermal synthesis method described by Kafshgari et al. in 2018,^63^ as shown in Figure 2a. In our experiment, we dissolved Fe(NO_3_)_3_-9H_2_O and Mn(NO_3_)_2_-4H_2_O in deionized water, creating an aqueous solution (metal salts were purchased from Sigma-Aldrich). The two solutions were mixed and stirred using a hot plate with a magnetic stirrer until complete dissolution of the reactants. While stirring, a NaOH solution was gradually added dropwise until reaching a desired pH, and the reaction temperature was maintained at 70 °C for 1 h. Subsequently, the resulting solution was transferred to a Teflon-lined hydrothermal autoclave reactor. The reactor was placed in an oven and heated at 180 °C for 16 h to promote the magnetic core formation. Once the reaction time was completed, the product was cooled to room temperature. To remove any impurities, the mixture was washed multiple times with absolute ethanol via magnetic decantation and dried in an oven to eliminate any remaining ethanol present. Next, the material was ground to a fine powder using an agate mortar, and 735.8 mg of dark brown magnetic powder was obtained. Finally, the MnFe_2_O_4_ magnetic powder was calcined at 600 °C for 6 h under UHP N_2_ inert atmosphere.

#### 2.1.2 Photoactive Material Growth by ALD

The obtained fine powder, described above, was coated with a ZnO layer by means of the ALD technique, as shown in Figure 2b. For the ALD coating, the used precursors were diethylzinc (DEZ) as organometallic precursors at room temperature (Growth Per Cycle, GPC = 1.8 Å/c) and deionized water as a reactant agent.^38,61^ The ALD process was carried out in a Beneq model TFS 200 system, with a reactor temperature of 150 °C. N_2_, purified to <10^–12^ ppm of O_2_, was utilized as carrier/purging gas during the entire process. The experimental setup has been documented in our previous works.^38^ In this case, 20 mg of magnetic powder was placed on a metal plate. On the rear of the plate, there is a matrix of nine Sm–Co permanent magnets, 5 mm in diameter, to avoid the magnetic material being pulled out by the mechanical pump to the outside of the ALD reactor during the coating process. Additionally, Si (100) substrates were placed within the reactor and on the metal plate as control samples to measure the layer thickness via the ellipsometry technique. The plate was situated at the entry point for precursors into the reactor chamber, where the ALD process was executed over 200 cycles, resulting in a coating thickness of approximately 36 nm, as estimated from ellipsometry. The ALD process to prepare the ZnO layer consists of the following sequence of pulses: 1 pulse of the DEZ precursor with 25 ms dose and 1 pulse of the H_2_O DI with 50 ms dose per cycle, with a purge time of 3 s for both precursors. The sequence described above is repeated 200 times to obtain the desired thickness described above. As mentioned earlier, the thicknesses for the ZnO coat were obtained by spectroscopic ellipsometry at room temperature by taking measurements on the Si control sample. Also, the MnFe_2_O_4_@ZnO nanocomposite was calcined under the same temperature, annealing time, and N_2_ inert atmosphere.

Figure 2 depicts the nanocomposite fabrication scheme based on MnFe_2_O_4_ magnetic cores coated with ZnO as the photoactive material.

In this study, we used atomic layer deposition (ALD) for coating MnFe_2_O_4_ magnetic nanoparticles due to its ability to provide precise thickness control, excellent uniformity, strong adherence, and high-purity ZnO films. These characteristics make ALD the preferred technique when ultrathin and high-quality coatings are required. Several deposition techniques, including magnetron sputtering, laser ablation, and spray pyrolysis,^64,65^ have been employed for ZnO thin film synthesis. However, sol–gel and hydrothermal synthesis methods are commonly used since they are cost-effective and easy to implement.^65^ Despite these advantages, sol–gel and hydrothermal approaches present significant challenges in achieving homogeneous ultrathin films, particularly on complex nanostructured surfaces.^66,67^ Moreover, only a few studies have reported successful deposition of ZnO ultrathin films onto MnFe_2_O_4_ magnetic nanoparticles in powder form using sol–gel or hydrothermal synthesis. In contrast, ALD is a widely used technique for depositing conformal ultrathin films on powdered materials, including magnetic nanoparticles with a high surface area and complex geometries, with promising applications in the photocatalysis field. Additionally, ZnO films synthesized via the sol–gel method typically require a postdeposition thermal treatment to achieve crystallization.^68^ On the other hand, the hydrothermal synthesis process can induce crystallization during the reaction, although in some cases, an additional thermal treatment enhances the material’s properties. In contrast, the ALD technique enables the deposition of ZnO films in the crystalline phase at a reactor temperature of 200 °C, eliminating the need for a postdeposition thermal treatment.^38,69^

#### 2.1.3 AZO Dye Preparation

**Figure 3:**
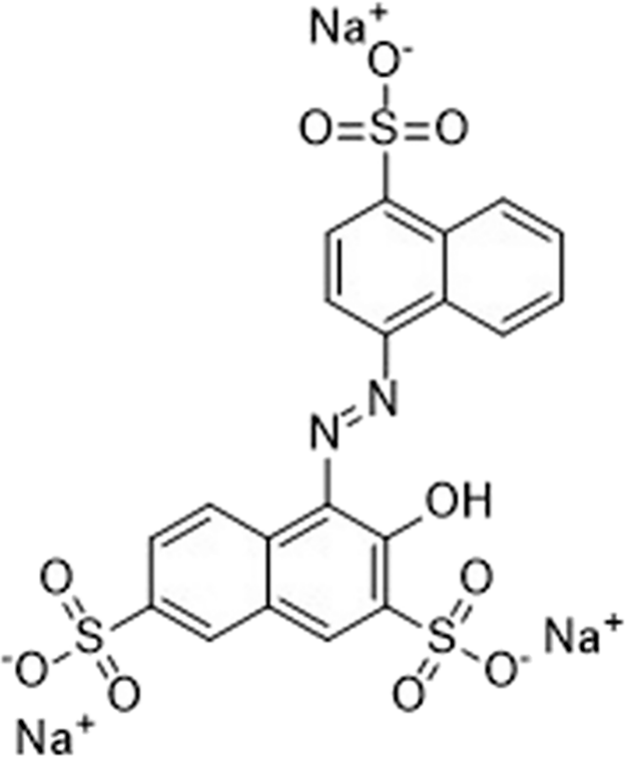
Organic molecule of Red Amaranth scheme^70^ (also known as E123) with empirical formula C_20_H_11_N_2_Na_3_O_10_S_3_ and characterized by AZO Group (−N=N−), Aromatic Rings, Sulfonic Acid Groups (−SO_3_H) and Sodium Salt. This figure was adapted from the National Center for Biotechnology Information (2025), PubChem Compound Summary for CID 13506, Amaranth. Retrieved April 15, 2025, from https://pubchem.ncbi.nlm.nih.gov/compound/Amaranth.

The dye used in this work was Red Amaranth, from Sigma-Aldrich (CAS number: 915-67-3), and empirical formula C_20_H_11_N_2_Na_3_O_10_S_3_. Red Amaranth is valued for its ability to produce intense reddish tones, making it a popular choice for coloring textiles, food products, cosmetics, and pharmaceuticals.^7,70^ Its AZO-bond (−N=N−) (see Figure 3) gives it stability and bright coloring facts, which have contributed to its extensive use in the industry. However, despite its widespread application, Red Amaranth has raised significant health and environmental concerns. Classified as a potentially toxic dye, it has been linked to various adverse effects on human health.^7^ For the photocatalytic experiment, the Red Amaranth dye solution was prepared and placed in a cylindrical quartz cell inside the reactor with a total volume of 250 mL at a concentration of 20 ppm.

### 2.2 Numerical Simulations

The finite-difference time-domain (FDTD) simulator from commercial Lumerical research software was utilized to model the scattering, absorption, and extinction efficiencies of MnFe_2_O_4_ and MnFe_2_O_4_@ZnO nanoparticles. The FDTD method operates by discretizing time-dependent Maxwell’s equations in both space and time, utilizing central differences to approximate partial derivatives. The shape and size of the nanoparticles were determined through TEM studies, while their optical indices were sourced from previous studies.^71,72^ A total-field scattered-field (TFSF) light source was employed, covering a wavelength range from 250 to 800 nm. To achieve high accuracy, a grid resolution of 0.2 nm was used. The absorption cross-section was calculated by positioning a cross-sectional analysis group within the TFSF source, whereas the scattering cross-section was determined using a similar analysis group placed outside the source. The extinction efficiency was subsequently obtained as the sum of the scattering and absorption efficiencies. To analyze the electric field distribution, a frequency-domain field monitor was positioned around the TFSF source. The surrounding medium was assumed to be water, with a refractive index of 1.33. The simulation region was enclosed by a Perfectly Matched Layer (PML) to minimize boundary reflections and ensure accurate results.

### 2.3 Characterization

The produced magnetic nanoparticles (NPs), with and without a ZnO coating, were subjected to various characterization techniques to obtain detailed information on their size distribution, morphology, crystalline structure, magnetic response, and photodegradation capacity of the Red Amaranth dye.

Morphological properties and size distribution for magnetic NPs were recorded in a JEOL JEM-2100F high-resolution transmission electron microscope (STEM) using an accelerating voltage of 200 and 400 kV, respectively. This microscope is equipped with an energy-dispersive X-ray spectroscopy (EDS) module for chemical mapping analysis. For TEM measurements, a small amount of fine powder was dispersed in ethanol to minimize the magnetic aggregates present in the carbon-coated copper grid (400 mesh). A small portion of the solution was dropped onto the copper grid and dried at room temperature before the characterization process.

The textural properties were characterized through N_2_ physisorption analysis by using a Micromeritics Tristar-II 3020 physisorption analyzer. The samples were pretreated under vacuum at 300 °C for 3 h using a sample degassing system (VacPrep-061) from Micromeritics Instruments. The textural properties were calculated from adsorption–desorption isotherm data, measured within the relative pressure (*P*/*P*_0_) range of 10^–5^–0.995. The specific surface area, pore volume, and pore size distribution of each solid were determined by using the classical Brunauer–Emmett–Teller (BET) method (Sing, 2014) and the Barrett–Joyner–Halenda (BJH) method (Barrett, Joyner, and Halenda, 1951).

The crystalline structure of the studied samples was obtained using a PANalytical X’Pert PRO diffractometer (CuK_α_ radiation with λ_CuKα_ = 1.5418 Å). Before characterizing, the magnetic photocatalyst nanocomposite was annealed at 600 °C for 3 h under a nitrogen atmosphere to avoid oxidation and subsequent formation of undesired crystalline phases. The diffractogram was registered in the 2θ range 10°-80° using a step size and dwell time of 0.02° and 0.5 s, respectively, and the patterns were compared to the ICSD (Crystal Structure Database) from HighScore Plus. Finally, from X-ray diffraction patterns, the structural parameters and crystal size for magnetic cores (MnFe_2_O_4_) and magnetic nanocomposite MnFe_2_O_4_@ZnO were determined.

The magnetic response of the MnFe_2_O_4_ and MnFe_2_O_4_@ZnO nanoparticle samples in dry powders was evaluated by vibrating sample magnetometry -VSM (MicroSens MicroSense EZ9HF), which allows determination of fundamental magnetic properties relevant for potential process applications, such as coercivity, saturation, and magnetic remanence. The hysteretic magnetization loop was measured at room temperature by varying the applied field between −20,000 and 20,000 Oe.

Thickness for the ZnO layers was obtained through spectroscopic ellipsometry (SE) at room temperature using a Spectroscopic Ellipsometer (VASE), J.A. Woollam Co. The required ellipsometric parameters (Ψ and Δ) for SE analysis were recorded in the wavelength spectral range from 240 to 1000 nm at four different incidence angles 45, 55, 65, and 75°. SE measurements were performed in the witness silicon substrates placed in the reactor and on the metallic plate located next to the magnetic powders.

Photodegradation experiments were achieved using magnetic nanoparticles coated with ZnO as photocatalysts to decompose the Red Amaranth dye. The dye solution was irradiated with UV–vis light, and the degradation process was monitored over time. Photodegradation efficiency was evaluated by comparing the performance of nanoparticles with and without the ALD coating. The Red Amaranth dye was subjected to photodegradation using a Rayonet photochemical reactor (model RPR 100), equipped with 16 lamps emitting at wavelength λ_uv_ = 250 nm. The experiment was accomplished with sampling intervals every 30 min until a total of 240 min. Dye degradation was assessed by measuring absorbance at 520 nm, corresponding to the characteristic absorption peak of this azo dye, in a Cary 50 UV–vis spectrophotometer at room temperature using a 4 mL quartz cuvette for sampling.

## 3 Results and Discussion

### 3.1 Structural Studies

**Figure 4:**
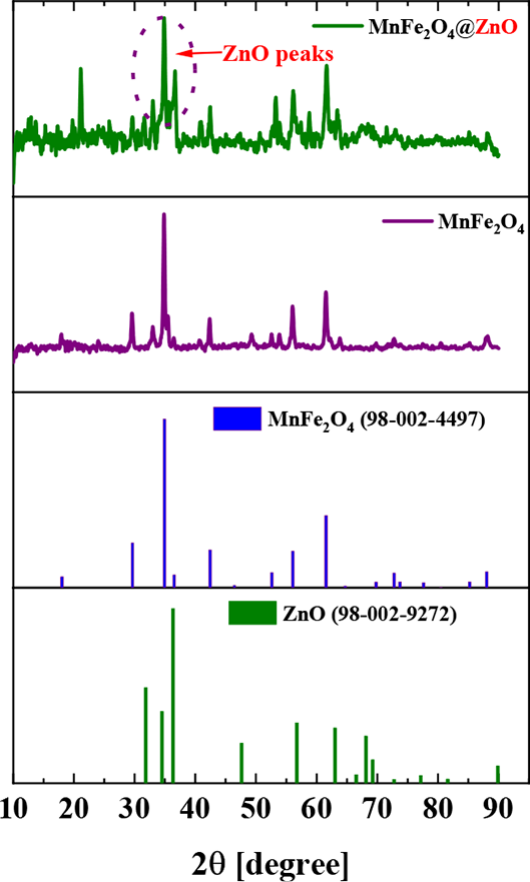
XRD pattern for MnFe_2_O_4_ magnetic nanoparticles and the MnFe_2_O_4_@ZnO nanocomposite.

An X-ray diffractogram is used to confirm nanoparticle crystal structure and crystallite size; Figure 4 presents XRD patterns of two samples: magnetic MnFe_2_O_4_ powder (purple line) and MnFe_2_O_4_@ZnO nanocomposite (green line). The diffractogram reveals distinct Bragg peak reflections at specific θ-2θ positions for both MnFe_2_O_4_ and MnFe_2_O_4_@ZnO. For MnFe_2_O_4_, characteristic Bragg peaks were observed at 17.93, 29.73, 32.88, 34.73, 42.85, 52.31, 55.98, 61.47, 72.77, and 88.76°, corresponding to the crystallographic planes (111), (220), (311), (222), (400), (422), (511), (440), (533), and (731). These reflections are consistent with a face-centered cubic (FCC) phase, typical of a normal spinel structure, and are indexed to the space group Fd3m, as corroborated by the ICSD reference file No. 98-002-4497.

In the MnFe_2_O_4_@ZnO nanocomposite, similar Bragg peak positions are observed at θ-2θ angles corresponding to the crystallographic planes of the MnFe_2_O_4_ magnetic core, confirming the presence of a normal spinel structure for manganese ferrite. In addition, new peaks appear, attributable to the wurtzite structure of ZnO (slightly overlapped with characteristic peak of spinel structure for magnetic cores), with reflections observed at θ-2θ positions 31.63, 34.91, 36.69, 47.41, 56.25, 63.37, 68.43, 72.95, 77.33, and 88.09°. These reflections correspond to the crystallographic planes (010), (200), (110), (210), (011), (310), (211), (400), (220), and (320) of ZnO. The wurtzite structure is confirmed by its space group P63mc, as indicated by ICSD reference file No. 98–002–9272. The presence of these ZnO peaks verifies the successful deposition of ZnO coating on the magnetic nanoparticles via ALD.

Using the Debye–Scherrer equation, the average crystallite size of the MnFe_2_O_4_ nanoparticles^73^ was estimated to be approximately 36.5 ± 0.2 nm, based on the XRD data. Furthermore, a shift in the XRD peaks toward higher diffraction angles is observed in the diffractograms of the powder materials. This shift, commonly referred to as a high-angle shift, indicates a reduction in crystallite size, which is typically induced by the high-temperature calcination process used during synthesis. As crystallite size decreases, the number of atoms per crystallite also diminishes, which subsequently affects the overall particle size of the powdered materials, also, the crystallite size is determined by factors such as the synthesis method, calcination conditions, and the intrinsic properties of the material.^74^ While a peak shift toward higher angles provides indirect evidence of reduced crystallite size, further complementary characterization techniques, such as TEM, are essential to fully elucidate the morphology and nanostructure of the fabricated materials.

### 3.2 Morphological Studies

**Figure 5:**
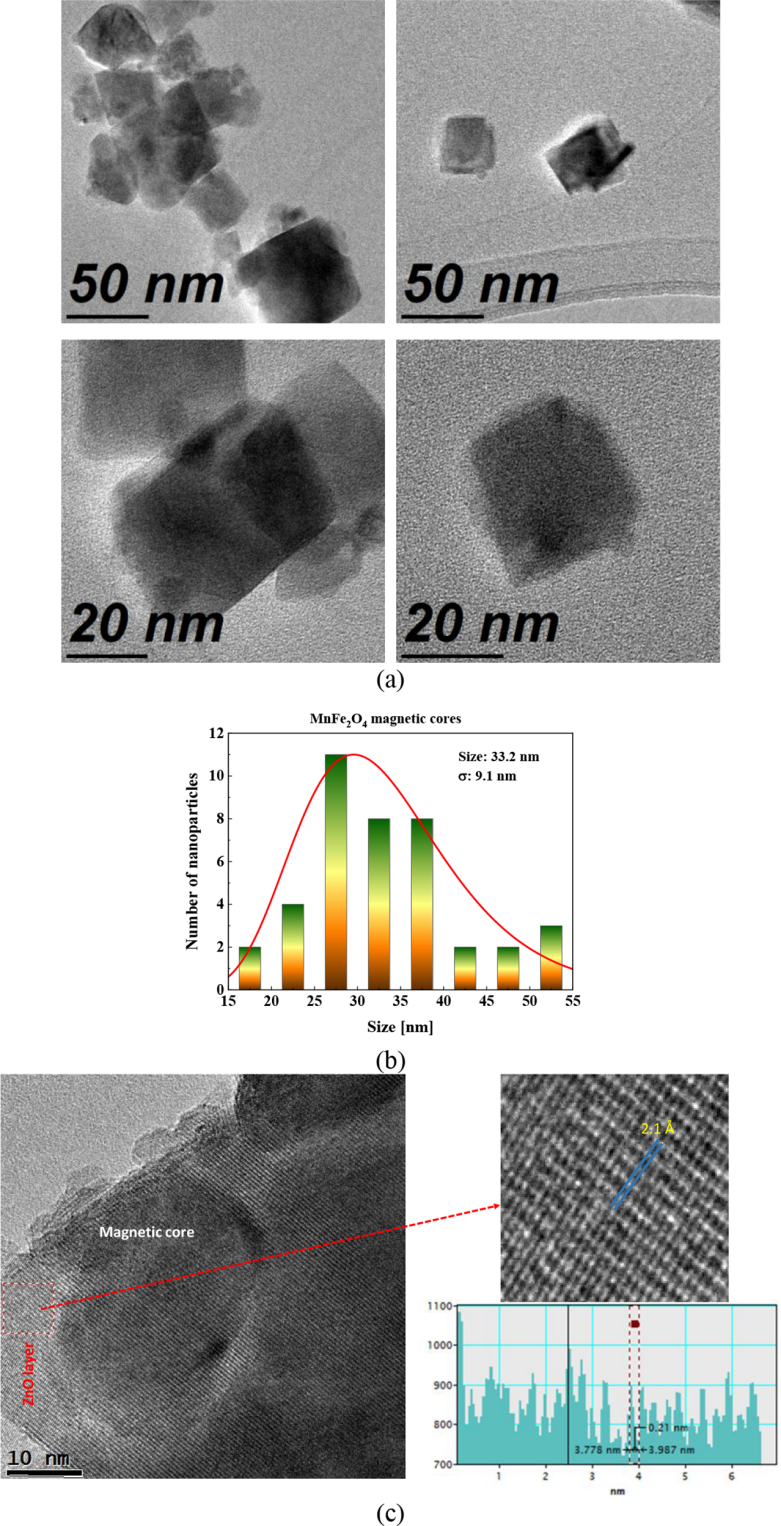
TEM and HR-TEM micrographs of MnFe_2_O_4_ magnetic nanoparticles and MnFe_2_O_4_@ZnO nanocomposite. (a) Micrographs show cubic morphology of studied nanoparticles at the 50 nm scale (above) and 20 nm scale (below). (b) Particle size distribution of MnFe_2_O_4_ cores before ZnO coating via ALD. (c) High-resolution and inset images (marked and highlight in red dashed lines) show crystalline zones of ZnO coating and well-defined crystalline structure of magnetic cores.

A TEM micrograph of MnFe_2_O_4_ and MnFe_2_O_4_@ZnO nanoparticles is presented in Figure 5. In Figure 5a, it is possible to observe well-defined grains agglomerated with cubic morphology, characterized by their uniform and consistent shape. The nanoparticle size was analyzed using ImageJ, an open-source software for processing and analyzing scientific images, revealing that the magnetic cores without a ZnO layer have an average size of around 33.2 ± 0.2 nm. The size distribution was fitted using a log-normal distribution function type, as shown in Figure 5b. In addition, Figure 5c exhibits small monocrystalline domains in the magnetic cores, indicating the high crystallinity present in the material. Also, there are crystalline zones associated with the ZnO film growth via ALD. The inset figure at the right of Figure 5c shows the inverse fast Fourier transform (IFFT) calculated from HR-TEM images of the region highlighted by red squares. IFFT results allowed us to obtain information about the planar distance, diameter, and crystallinity degree for the sample. A continuous, black and bright, well-defined fringe can be observed, indicating the highly crystalline nature of the NPs and the ZnO film.

According to these analyses, the hydrothermal method allows us to obtain MnFe_2_O_4_ magnetic nanoparticles with well-defined cubic geometry. Therefore, it was chosen to fabricate magnetic nanoparticles due to their ability to yield uniform and dispersed nanoparticles with controlled morphology. It enables precise regulation of reaction parameters such as temperature, pressure, and reaction time, thereby facilitating the production of high-purity materials and well-defined particle sizes, typically within the nanometer to micrometer range. In addition, the presented morphological and size results are in good agreement with DRX crystallite size and magnetic behavior studies from the hysteresis loop, confirming that the magnetic NPs could be considered a soft magnetic material, which is easily magnetized and demagnetized under an external magnetic field.

**Figure 6:**
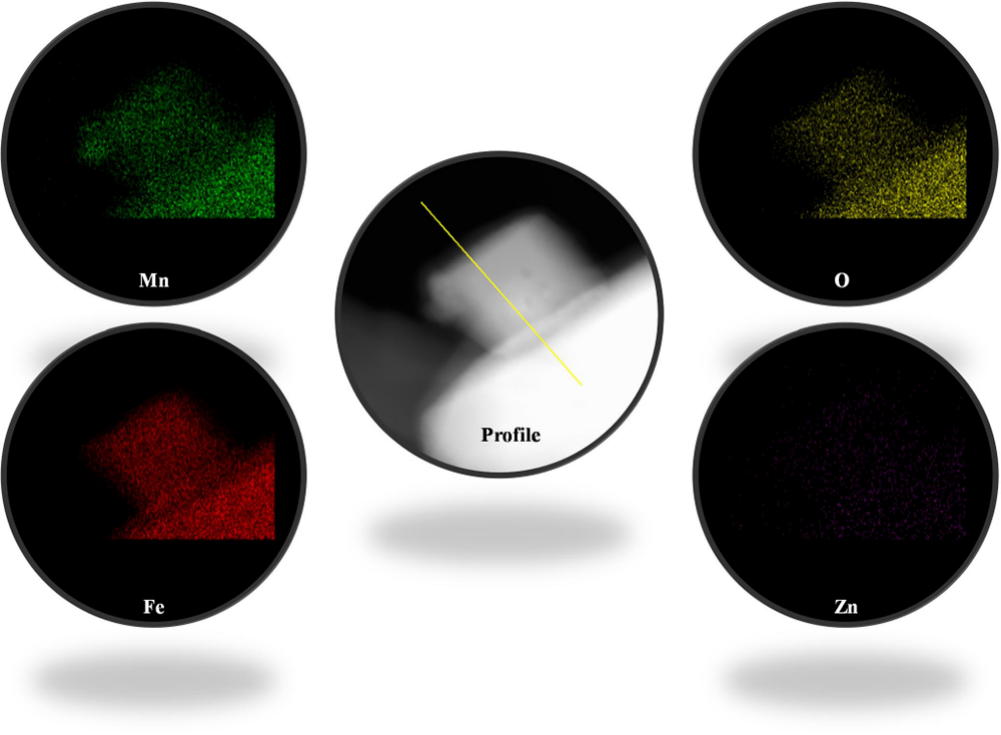
Chemical mapping of the MnFe_2_O_4_@ZnO nanocomposite. The STEM-EDS elemental maps illustrate the spatial distribution of the constituent elements within the material. The central image represents the region from which the STEM-EDX spectra were acquired. The elemental distribution is depicted using false-color representation as follows: Mn (green), Fe (red), O (yellow), and Zn (violet).

The presence of cubic-shaped grain aggregates indicates effective control over particle nucleation in the fabrication process, demonstrating that the synthesis of tailored nanostructures was designed successfully. This precise control over morphology and particle distribution could enhance nanocomposite performance in various technological applications, such as in heterogeneous photocatalysis, which involves magnetic materials coated with semiconductor films for environmental remediation. Additionally, Figure 6 presents STEM-EDS elemental mapping of MnFe_2_O_4_@ZnO nanocomposite, displaying a pseudocolor image generated from transmitted electrons corresponding to nanoparticles along the selected profile line scan (shown in the central image). The green color represents the X-ray emission from Mn–K, indicating the distribution of manganese atoms within the magnetic core. The red color corresponds to Fe K emission, revealing the presence of iron within the nanoparticles. The yellow color indicates the emission of O–K, associated with oxygen in the ferrite, while the violet color represents Zn–K emission, reflecting the distribution of zinc atoms on the magnetic nanoparticles, deposited via the ALD method. This elemental analysis demonstrates a uniform distribution of transition metals and the photoactive ZnO material throughout the MnFe_2_O_4_@ZnO nanocomposite, synthesized via the hydrothermal method and subsequently coated with ZnO. This consistent elemental distribution confirms the successful fabrication of the nanocomposite material.

### 3.3 BET Measurement

The surface area of a photocatalyst is a critical parameter that significantly influences its photodegradation performance as it directly affects the availability of active sites for photocatalytic reactions. Table 1 relates the textural and surface properties of the materials (specific surface area (*A*_BET_), pore volume, and average pore size) obtained by N_2_ physisorption analysis. As is appreciated, *A*_BET_ decreases from 35.6 m^2^ g^–1^(MnFe_2_O_4_) to 16.2 m^2^ g^–1^ (MnFe_2_O_4_@ZnO), pore volume from 0.101 cm^3^ gr^–1^ (MnFe_2_O_4_) to 0.069 cm^3^ gr^–1^ (MnFe_2_O_4_@ZnO), and average pore size from 21.2 nm (MnFe_2_O_4_) to 16.2 nm (MnFe_2_O_4_@ZnO). This considerable decrease in the textural and surface properties of the MnFe_2_O_4_@ZnO photocatalyst is attributed to the ZnO thin film deposited on magnetic-supported material, as has been confirmed by TEM characterization.

**Table 1:** Textural Properties (BET Specific Surface Area, Pore Volume, and Average Pore Size) of a Magnetic Photocatalyst Nanocomposite Based on MnFe_2_O_4_@ZnO

catalyst	BET surface area [m^2^ g^–1^]	pore volume [cm^3^ g^–1^]	pore size [nm]
MnFe_2_O_4_	35.6	0.101	21.2
MnFe_2_O_4_@ZnO	16.2	0.069	14.2

### 3.4 Magnetic Properties

**Figure 7:**
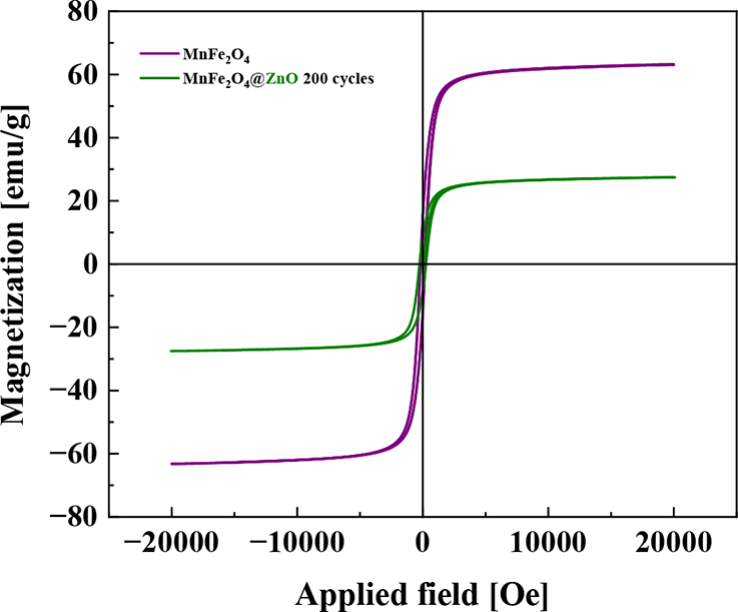
M vs H hysteresis loop at room temperature for MnFe_2_O_4_ magnetic nanoparticles and the MnFe_2_O_4_@ZnO nanocomposite material.

Magnetization (*M*) as a function of the magnetic field (*H*) measurements at room temperature for MnFe_2_O_4_ nanoparticles and MnFe_2_O_4_@ZnO nanocomposite are shown in Figure 7. A small hysteresis loop is observed, with saturation magnetization (*M*_s_) values of 63 emu g^–1^ for MnFe_2_O_4_ and 27 emu g^–1^ for MnFe_2_O_4_@ZnO, along with coercivity (*H*_c_) values of 186 and 260 Oe, respectively. The coercivity being close to zero relative to the applied external magnetic field indicates that the MnFe_2_O_4_ nanoparticles exhibit characteristics of a soft magnetic material. This implies that the material can be easily magnetized and demagnetized, as evidenced by its low coercive field and the increase in magnetization until saturation is reached. The low coercivity observed suggests that both MnFe_2_O_4_ and MnFe_2_O_4_@ZnO nanoparticles behave similarly to superparamagnetic materials. This behavior is characterized by a significant number of magnetic moments aligning with the external magnetic field, contributing to the net magnetization of the material in the presence of an applied field. Assuming that the fabricated magnetic nanoparticles are monocrystalline, the magnetic properties of MnFe_2_O_4_ can be correlated with particle and crystallite sizes, which were determined through XRD and TEM. The relationship between particle size and magnetic properties indicates a tendency toward superparamagnetic behavior characterized by reduced coercivity and enhanced saturation magnetization. This correlation is crucial for applications requiring precise control over the magnetic properties of nanoparticles, in this case, the magnetic separation process in wastewater environmental remediation.

### 3.5 Photodegradation Test

**Figure 8:**
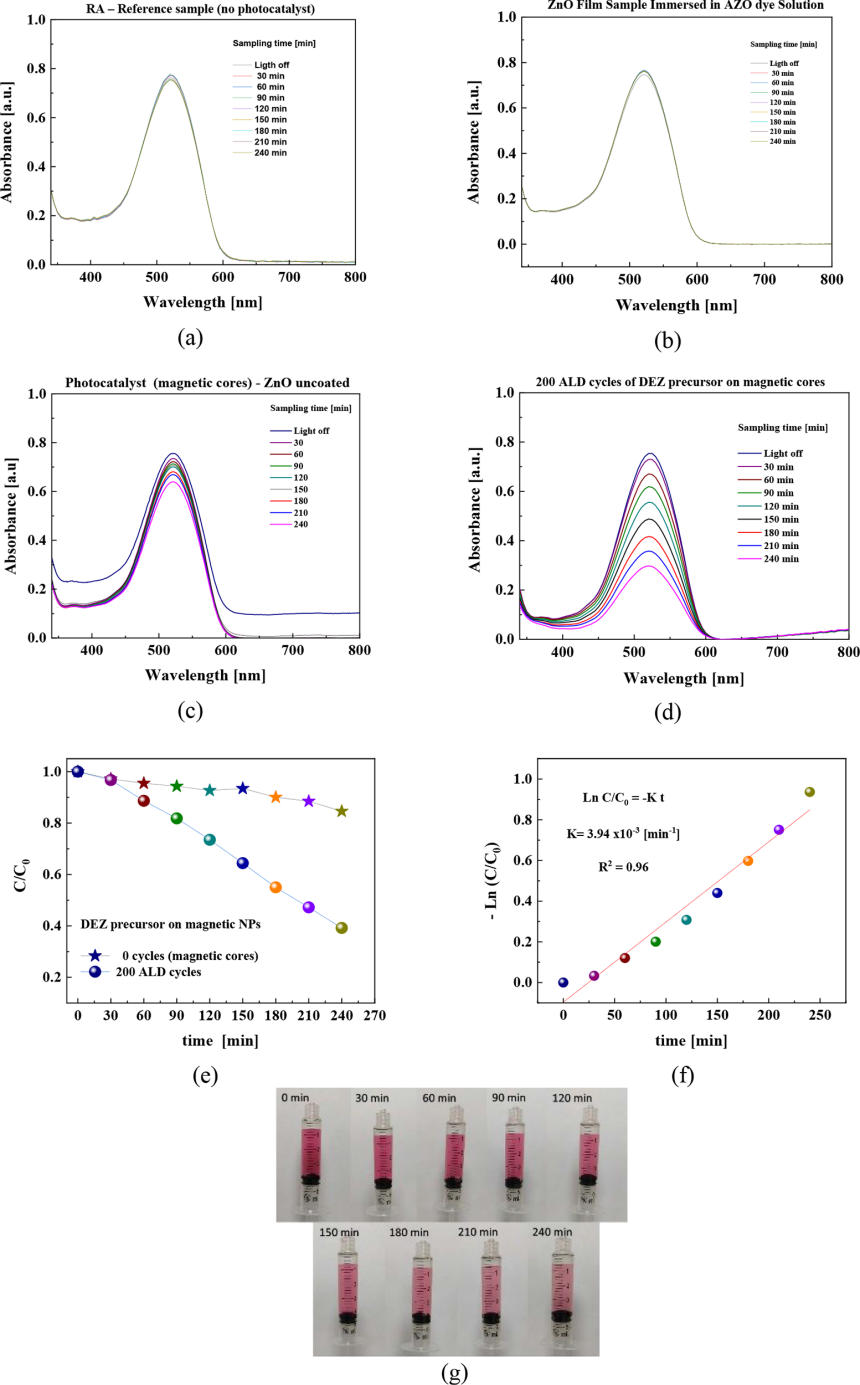
Absorbance spectra for MnFe_2_O_4_@ZnO (a) RA control sample without photocatalyst, (b) ZnO film sample immersed in AZO dye solution, (c) magnetic nanoparticles without ZnO coating, and (d) magnetic nanoparticles coated with ZnO by ALD. (e) Normalized absorbance spectra under UV–vis radiation with the photocatalyst synthesized from the magnetic nanoparticles, (f) photodegradation kinetics of Amaranth dye, (g) gradual color fading for Red Amaranth dye up to 240 min of exposure.

After coating the magnetic cores with 200 cycles of DEZ precursor to generate an ultrathin film of ZnO, the photocatalytic activity was tested by monitoring the Red Amaranth dye concentration every 30 min by examining the intensity of the absorbance peak at 520 nm with a UV–visible spectrophotometer, as shown in Figure 8, to observe the photodegradation process. Figure 8a–d corresponds to the control sample (only AZO dye without photocatalyst), ZnO film sample immersed in AZO dye solution, MnFe_2_O_4_ magnetic cores without the ZnO layer, and the magnetic cores surface coated with ZnO layer via ALD. By comparing these plots, there is an evident peak intensity decrease as time passes during sampling for the sample with the ZnO layer. However, the sample without the ZnO layer on the magnetic cores presents a negligible intensity loss after 240 min under UV irradiation compared with the control sample (Red Amaranth without photocatalyst). To assess the influence of the ZnO layer on the photodegradation process, the ultrathin film deposited on the silicon substrate used as a witness and placed in the ALD reactor during the coating of the magnetic cores process was placed into the quartz cuvette with AZO dye. Following this, the photocatalysis experiment was performed under identical experimental conditions (sampling time, power UV light, etc.), enabling a direct comparison of its photocatalytic performance with that of the composite material.^75^ The results of this experiment indicated that just the ZnO film in contact with the dye did not exhibit a significant effect on dye degradation compared with the impact on the dye due to the ZnO layer coating on the surface of magnetic cores. Figure 8e shows the normalized absorbance spectra under UV–vis radiation with the photocatalyst synthesized from the magnetic nanoparticles; the photodegradation continued over 4 h, resulting in a degradation efficiency η_eff_ around 60%.

The degradation efficiency was determined using the following relation:
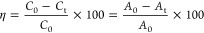
1where *A*_0_ corresponds to the initial absorbance for the dye mixture and *A*_t_ is the absorbance at t time, respectively; both values are related to the initial concentration, *C*_0_, as well as the concentration *C*_t_ at *t* time, following the Lambert–Beer law. The designed magneto-controlled photocatalyst successfully degraded Red Amaranth dye, which is a common pollutant in the textile and food industries. Photocatalytic degradation tests demonstrated the potential of this material for scalable applications in water remediation. The material is environmentally friendly, taking advantage of the magnetic properties of Manganese ferrite cores and the photoactive features provided by the ZnO ultrathin coating on the cores. Additionally, the magnetic core could enable the photocatalyst to be easily recovered from water by using an external magnetic field, facilitating its reuse via a magnetic separation process that can be implemented in industrial water-treatment processes.

**Figure 9:**
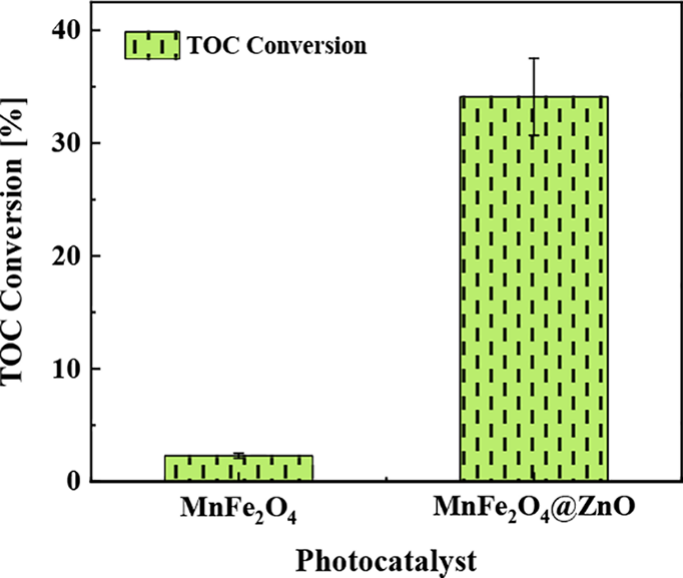
TOC (%) measured after the end of reaction (240 min) for MnFe_2_O_4_ magnetic nanoparticles and the MnFe_2_O_4_@ZnO nanocomposite material.

Photodegradation kinetics was evaluated using the equation
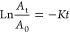
2where *A*_0_ and *A*_t_ denote the absorbance values before and after UV irradiation at time *t* during the photocatalytic process, respectively, and *K* [min ^–1^] is the rate constant. The *K* value was obtained by fitting the experimental data in Figure 8f using linear regression analysis based on eq 2. The *K* constant represents the reaction rate, and the photocatalytic kinetics determine the dye degradation rate, following a pseudo-first-order kinetic model. The corresponding *K* value for the decolorization process was approximately 3.94 × 10 ^–3^ min ^–1^, considering data every 30 min during 240 min under a UV–vis light source. Figure 8g demonstrates the systematic decolorization of Red Amaranth throughout the experiment, with the solution gradually transitioning from an intense red to progressively lighter shades. This color change serves as a visual indicator of the dye’s progressive degradation under photocatalytic conditions. The fading of color corresponds to the breakdown of the azo dye molecules as the active photocatalyst facilitates the reduction in dye concentration. Decolorization is particularly pronounced with an increasing irradiation time, confirming the effectiveness of the photocatalytic process. This visual representation highlights the nanocomposite fabricated efficiency in promoting dye degradation, further supported by quantitative data in Figure 8a–c**.** To evaluate the mineralization degree of Red Amaranth dye at the end of the reaction, the total organic carbon parameter was determined, which indicates the level of organic carbon converted to inorganic carbon (CO_2_). The results indicate (Figure 9) a TOC conversion of 2.3 and 34.1% for MnFe_2_O_4_ and MnFe_2_O_4_@ZnO, respectively. It is worth noting that the TOC conversion obtained with the MnFe_2_O_4_@ZnO photocatalyst is like or even above other studies previously reported where this dye is used as a model molecule, or analogous materials are employed to eliminate other dyes (see Table 2).

**Table 2:** Comparison of AZO Dye Degradation Using Different Photocatalysts: Correlation between Degradation Performance and the TOC Percentage

photocatalyst concentration		dye	degradation [%]	TOC [%]	time [h]	reference
TiO_2_ P-25/Cu	10 [mg/L]	O–II	97	89	9	(76)
Fe- TiO_2_	3 [g/L]	AO7	80	73	1	(77)
TiO_2_@Fe_3_O_4_	5 [mg/L]	MB	66.6	48.21	10	(78)
ZnO/MnFe_2_O_4_	5 [mg/L]	MB	85%	71.5	4	(79)
TiO_2_ thin films	no report	AY	40	74.46	24	(80)
TiO2 - ZrO2	10 [mg/L]	RhB	78.1	74.3	3	(81)
Sm-doped ZnO	236 [mg/L]	CR	84.5	no report	4	(82)
SnP@ZnO	50 [mg/L]	AM	41	78	1	(83)
ZnO	no report	AM	74	no report	7	(84)

The organic carbon content of the solution was determined by total organic carbon (TOC) measurement using a spectrometer (Hach DR2800, method 10129), and the TOC conversion was calculated according to the following equation:
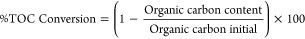
3

**Figure 10:**
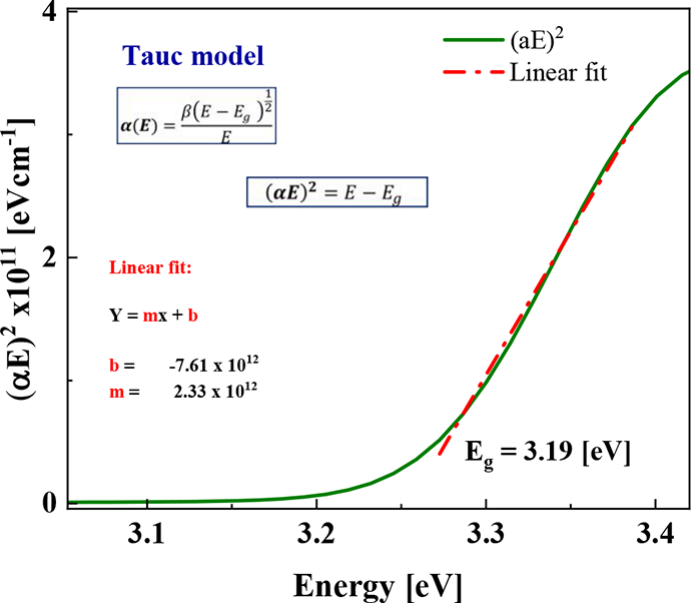
Estimation of the optical bandgap energy using the Tauc–Lorentz model.

In addition, the optical band gap of a photoactive material is a critical parameter in photodegradation tests, as it dictates the material’s ability to absorb light and generate charge carriers essential for photocatalytic reactions. In our study, the bandgap energy (*E*_g_) of the ZnO film deposited on magnetic cores was determined from absorbance data, obtaining a value close to 3.19 eV, calculated using the Tauc–Lorentz model, which is widely used for the optical characterization of semiconductors and dielectrics. The 3.19 eV bandgap value for ZnO is consistent with typical values reported in the literature, although it may vary slightly depending on morphology, synthesis method, and experimental conditions. It is well-known that the Tauc-Lorentz model combines Tauc’s equation, used to determine the bandgap in amorphous and polycrystalline semiconductors, with the Lorentz model, which describes the dielectric response. For a first-order transition (allowed direct transition), the relationship between the absorption coefficient α and the photon energy *h*ν*.*^85^Figure 10 depicts the band gap obtained using the Tauc model, in this case, to determine the 3.19 eV band gap value, (α*E*)^2^ was plotted as a function of *E*. A linear fit was applied to the linear region of the curve to obtain the slope and intercept values. Finally, this fitted line was extrapolated to the energy axis to determine the *E*_g_ value.

**Figure 11:**
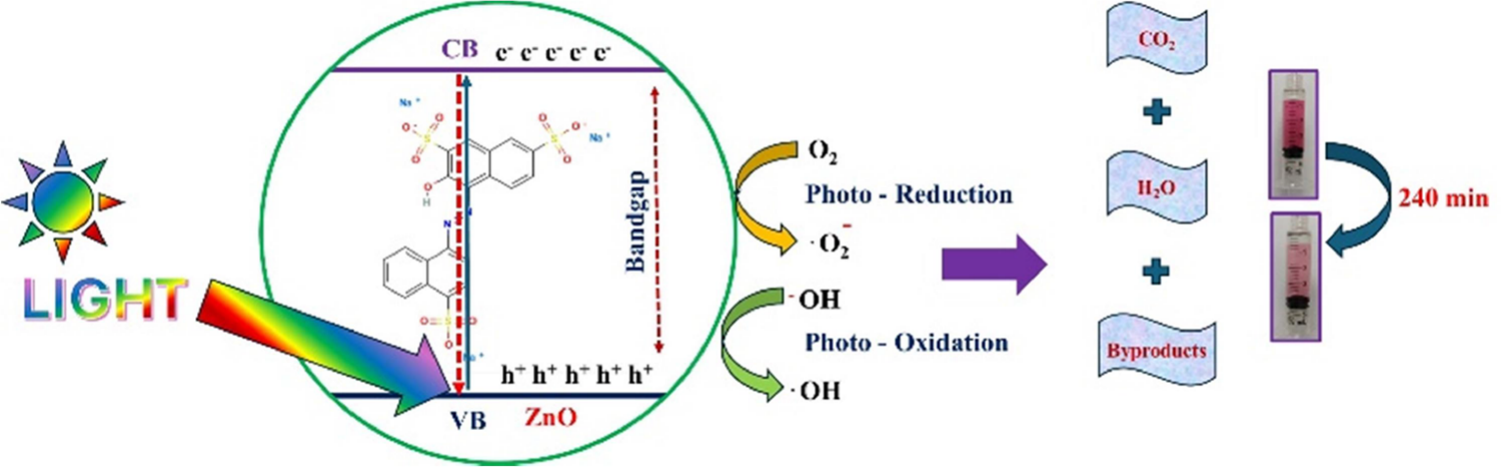
Photodegradation scheme process for the MnFe_2_O_4_@ZnO nanocomposite.

As discussed before, knowing the optical band gap value is essential for understanding the photocatalytic behavior of the ZnO layer. This is relevant in the photodegradation process, where the efficiency of light absorption directly influences charge carrier generation and reactive species formation. Photodegradation process schemes using the MnFe_2_O_4_@ZnO nanoparticles are shown in Figure 11. The photodegradation mechanism for our nanocomposite begins when light, from the source, is absorbed by the ZnO layer deposited on a magnetic nanoparticle, promoting electrons from the valence band to the conduction band. The transferred electrons reduce oxygen molecules to form superoxide anions (O_2_^–^), while the holes oxidize water or hydroxide ions to generate hydroxyl radicals (OH). These reactive species degrade organic pollutants like Amaranth dye into smaller molecules such as CO_2_ and H_2_O.^86,87^

### 3.6 Theoretical Approximation

**Figure 12:**
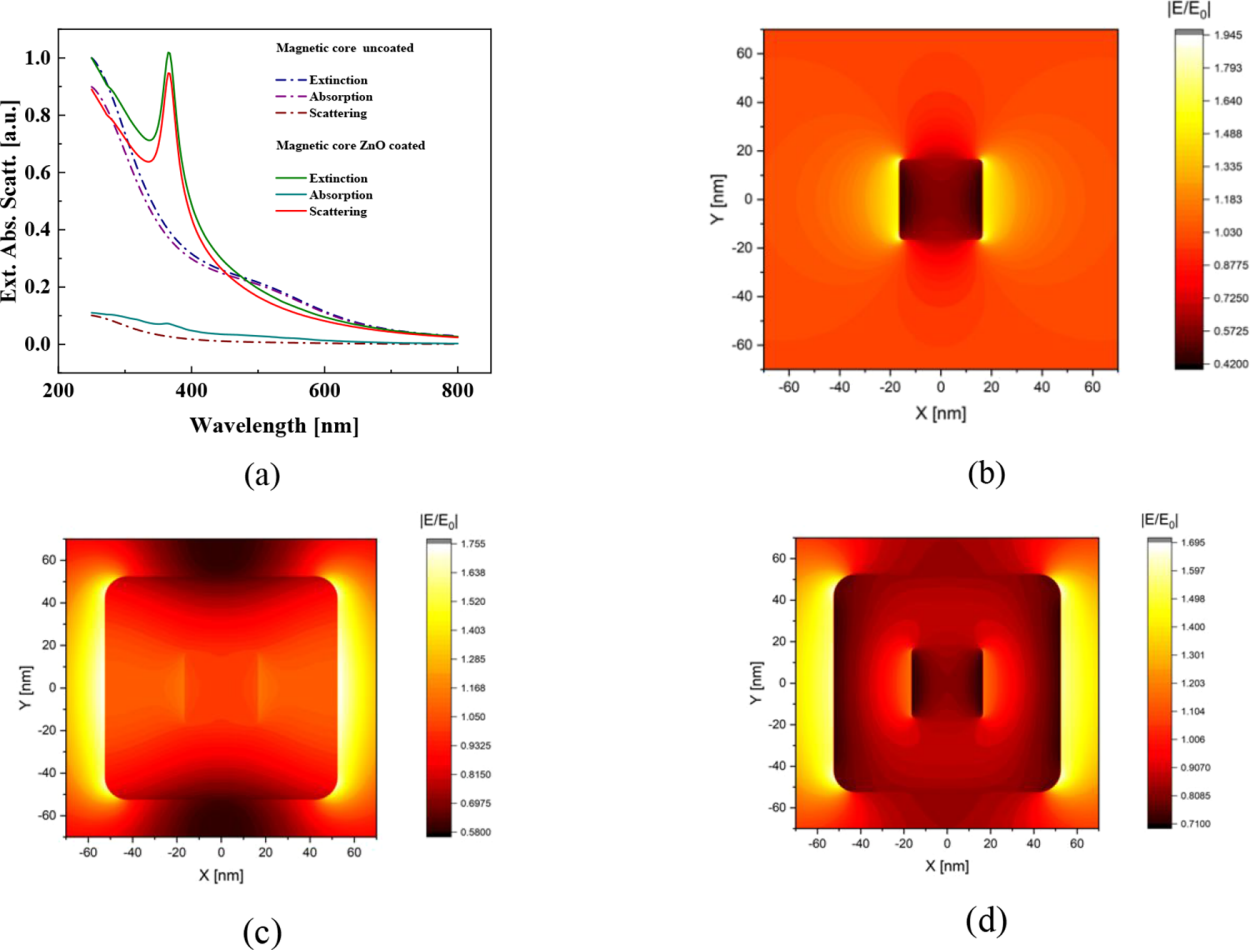
(a) Normalized extinction, scattering, and absorption spectra for the ZnO-coated (solid lines) and uncoated (dash-dot-dot lines) MnFe_2_O_4_ nanoparticles. (b) Near-field for a MnFe_2_O_4_ nanoparticle around 520 nm. (c) and (d) Near-fields for the ZnO-coated MnFe_2_O_4_ nanoparticles, calculated at 365 and 520 nm, respectively.

The enhanced photodegradation efficiency of ZnO-coated MnFe_2_O_4_ nanoparticles, compared to pure MnFe_2_O_4_ nanoparticles, can be qualitatively explained by examining numerical simulations of their absorbance and scattering cross sections, as shown in Figure 12a. The extinction cross-section, which combines absorbance and scattering, provides a numerical approximation of the experimental UV–vis absorbance measurements. In these calculations, performed with nanoparticles in water (refractive index = 1.33), all data are normalized to the maximum extinction value of the ZnO-coated MnFe_2_O_4_ nanoparticles. Results are presented with solid lines for ZnO-coated MnFe_2_O_4_ and dashed-dotted lines for uncoated nanoparticles. In uncoated nanoparticles, absorbance (associated with optical losses) is the primary factor determining extinction and exhibits a monotonic decrease, accompanied by a small shoulder (due to the optical resonance of the dielectric nanoparticle) around 500 nm. In contrast, for the coated nanoparticles, scattering (due to the strong dipole resonance of the nanocube) dominates along the entire spectrum of the study, with a sharp peak around 400 nm. To further understand the experimentally observed photodegradation, from an electromagnetic point of view, we simulated near-fields at wavelengths relevant to Red Amaranth strong absorption peak around 520 nm (Figure 8c,d). Figure 12b depicts the near-field for uncoated MnFe_2_O_4_ nanoparticles, revealing an enhanced dipole-like field around the nanoparticle surface. This field results from absorption due to optical losses in the MnFe_2_O_4_ material, which only modestly contributes to photodegradation, as shown in Figure 8c.

**Figure 13:**
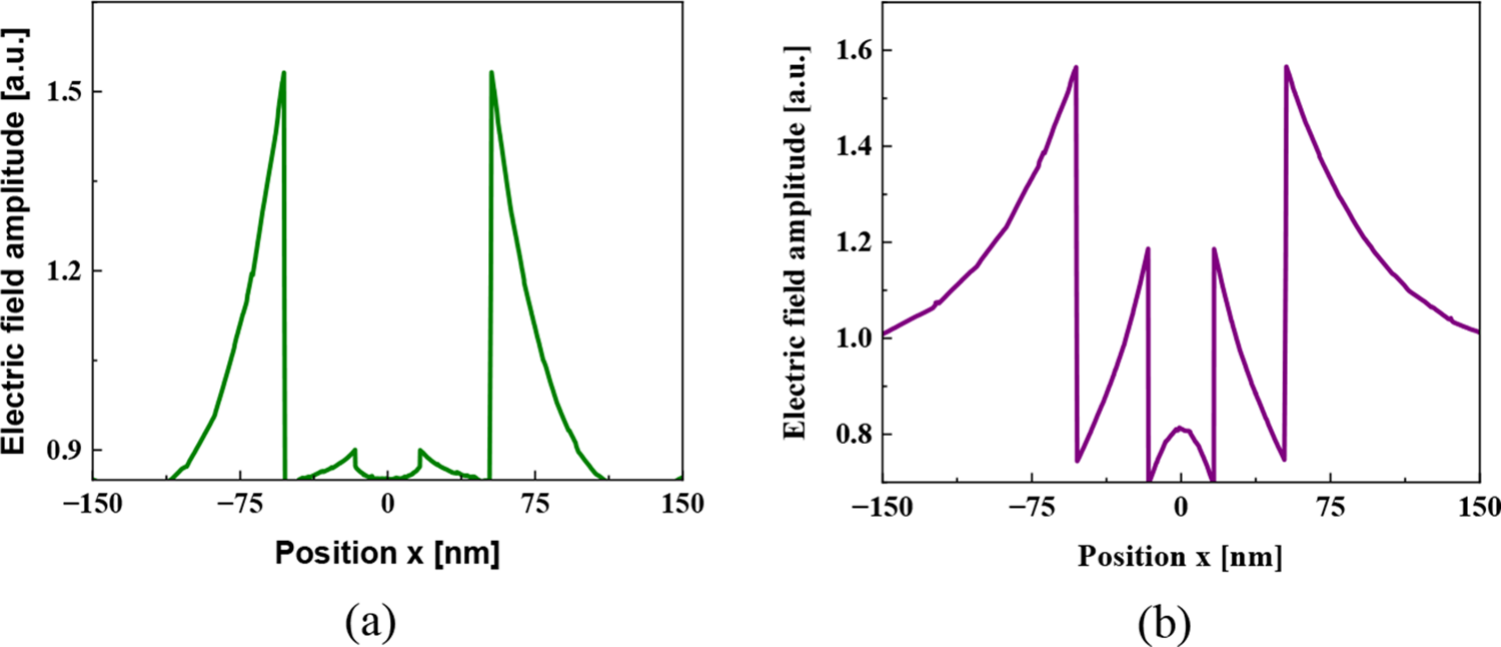
Normalized electric field profiles along the *xz*-plane for MnFe_2_O_4_@ZnO nanocubes calculated at (a) 365 and (b) 520 nm, respectively.

In the case of ZnO-coated MnFe_2_O_4_ nanoparticles, the near-field at 365 nm, shown in Figure 12c, demonstrates significant field enhancement due to light scattering associated with a strong dipole resonance. Additionally, Figure 12d shows the near field at 520 nm, where the enhanced and localized fields at the ZnO-medium interface contribute to a stronger light-matter interaction. These enhanced near-fields polarize the environment surrounding nanoparticles, along with the high absorption by the inner MnFe_2_O_4_ nanoparticles (see Figure 12c), are key factors that enhance the photodegradation performance of ZnO-coated MnFe_2_O_4_ nanoparticles in the degradation of Red Amaranth. This phenomenon is further illustrated in Figure 13a,b, which present the normalized electric field profiles along the *xz*-plane at the middle of the nanocubes. While the resonant electromagnetic field at 520 nm exhibits a slightly lower amplitude than at 365 nm, where the field is predominantly concentrated at the ZnO outer surface, it displays a more distributed nature. The 520 nm field extends across both the inner and outer interfaces of the ZnO-coated MnFe_2_O_4_ structure, exhibiting a slower decay with an increasing distance from the nanoparticle. Although the presence of interfering ions (not considered in this study) may hinder the degradation of certain pollutants,^80^ such effects cannot be fully captured by the electromagnetic simulations conducted here.

## 4 Conclusions

In this study, MnFe_2_O_4_ nanoparticles were synthesized using a hydrothermal method and subsequently coated with a ∼36 nm layer of ZnO via atomic layer deposition, forming MnFe_2_O_4_@ZnO as a photoactive material. A detailed examination of their structural, morphological, and magnetic properties, along with their photodegradation performance, was conducted. Crystallographic analyses confirmed the formation of a spinel structure associated with manganese ferrite magnetic cores. Additionally, a hexagonal wurtzite phase, characteristic of the ZnO coating, was observed on the MnFe_2_O_4_@ZnO samples. The crystallite size of the magnetic nanoparticles was determined to be 36.5 ± 0.2 nm, while the overall nanoparticle size was measured to be 33.2 ± 9.1 nm. Comparative analysis showed that both the uncoated and ZnO-coated nanoparticles exhibited a tendency toward superparamagnetic behavior at room temperature, as indicated by a minor hysteresis loop. However, the ZnO-coated magnetic cores displayed a reduction in saturation magnetization (in emu/g), attributed to the presence of the ZnO semiconductor layer. It can be asserted that the MnFe_2_O_4_@ZnO nanocomposite-based photocatalyst is a multifunctional material, as it combines the photocatalytic activity provided by the ALD-deposited ZnO coating with the magnetic response of the nanoparticles. Our study demonstrated that the photocatalytic and magnetic properties of this system were effective in degrading Red Amaranth azo dye, highlighting the potential to explore various magnetic materials coated with photoactive layers using the ALD technique. The results showed that the ZnO coating on the magnetic cores achieved a degradation efficiency of nearly 60% for the toxic Red Amaranth dye solution after 240 min. Finite-difference time-domain (FDTD) electromagnetic simulations were conducted to further support these findings. Overall, the results indicate that the MnFe_2_O_4_@ZnO nanocomposite can be classified as a soft magnetic and multifunctional material, with significant potential for photocatalytic applications combined with magnetic separation processes, offering an eco-friendly alternative for wastewater treatment.

## Data Availability

The data supporting this study’s findings are presented throughout the manuscript. The corresponding data can be visualized in OriginPro 2023 by clicking on each graph. To clarify, this study does not include additional supplementary files. Further details regarding the experimental procedures and analytical methods can be provided by the corresponding author, J.A.L.M., upon reasonable request.
